# Development of a Novel [^11^C]CO-Labeled Positron Emission Tomography Radioligand [^11^C]BIO-1819578 for the Detection of *O*-GlcNAcase Enzyme Activity

**DOI:** 10.1021/acschemneuro.3c00247

**Published:** 2023-06-28

**Authors:** Sangram Nag, Martin Bolin, Prodip Datta, Ryosuke Arakawa, Anton Forsberg Morén, Yasir Khani Maynaq, Edward Lin, Nathan Genung, Heike Hering, Kevin Guckian, Laurent Martarello, Maciej Kaliszczak, Christer Halldin

**Affiliations:** † Department of Clinical Neuroscience, Center for Psychiatry Research, Karolinska Institutet and Stockholm County Council, Stockholm 17176, Sweden; ‡ BIOGEN MA Inc., 225 Binney St., Cambridge, Massachusetts 02142, United States

**Keywords:** PET, OGA, radiolabeling, non-human primate, radiometabolites, in vivo

## Abstract

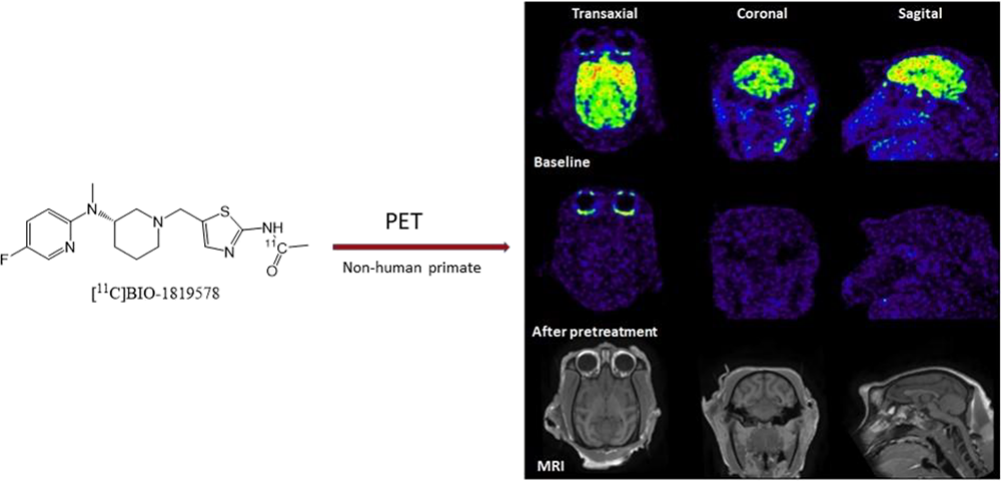

Imaging *O*-GlcNAcase OGA by positron emission tomography (PET) could provide information on the pathophysiological pathway of neurodegenerative diseases and important information on drug-target engagement and be helpful in dose selection of therapeutic drugs. Our aim was to develop an efficient synthetic method for labeling BIO-1819578 with carbon-11 using ^11^CO for evaluation of its potential to measure levels of OGA enzyme in non-human primate (NHP) brain using PET. Radiolabeling was achieved in one-pot via a carbon-11 carbonylation reaction using [^11^C]CO. The detailed regional brain distribution of [^11^C]BIO-1819578 binding was evaluated using PET measurements in NHPs. Brain radioactivity was measured for 93 min using a high-resolution PET system, and radiometabolites were measured in monkey plasma using gradient radio HPLC. Radiolabeling of [^11^C]BIO-1819578 was successfully accomplished, and the product was found to be stable at 1 h after formulation. [^11^C]BIO-1819578 was characterized in the cynomolgus monkey brain where a high brain uptake was found (7 SUV at 4 min). A pronounced pretreatment effect was found, indicating specific binding to OGA enzyme. Radiolabeling of [^11^C]BIO-1819578 with [^11^C]CO was successfully accomplished. [^11^C]BIO-1819578 binds specifically to OGA enzyme. The results suggest that [^11^C]BIO-1819578 is a potential radioligand for imaging and for measuring target engagement of OGA in the human brain.

## Introduction

Alzheimer’s disease (AD) is the most common cause of dementia. AD is characterized by the accumulation of neurofibrillary tangles, fibrillary lesions in the form of neurotic plaques, and dystrophic neurites, which contain hyperphosphorylated, insoluble intracellular tau protein.^1^ The symptoms and progress of AD are strongly related to the magnitude and location of pathological intracellular tau.^2^ O-GlcNAcylation is a common post-transcriptional glycosylation where intracellular proteins can be modified by single-sugar O-linked-β-*N*-acetyl-glucosamine (O-GlcNAc). This modification is regulated by the specific enzymes, O-GlcNAc transferase (OGT) and 3-*O*-(*N*-acetyl-d-glucosaminyl)-l-serine/threonine *N*-acetylglucosaminyl hydrolase (OGA).^3^ OGA catalyzes the hydrolysis of O-linked β-*N*-acetyl glucosamine (O-GlcNAc) modification and thereby modulates the function of numerous cellular proteins.^4^ It is reported that tau O-GlcNAc modification is increased through the inhibition of OGA, which markedly reduces tau aggregation but does not prevent tau hyperphosphorylation.^5^ Enzymatic activity of OGA has been implicated not only in AD but also in other diseases, for example, stress,^6^ Parkinson’s and Huntington’s diseases,^7^ and type 2 diabetes.^8^ Because of their therapeutic importance, several selective OGA inhibitors have entered clinical trials with the most advanced undergoing phase 2 clinical studies.^9^

**Figure 1:**
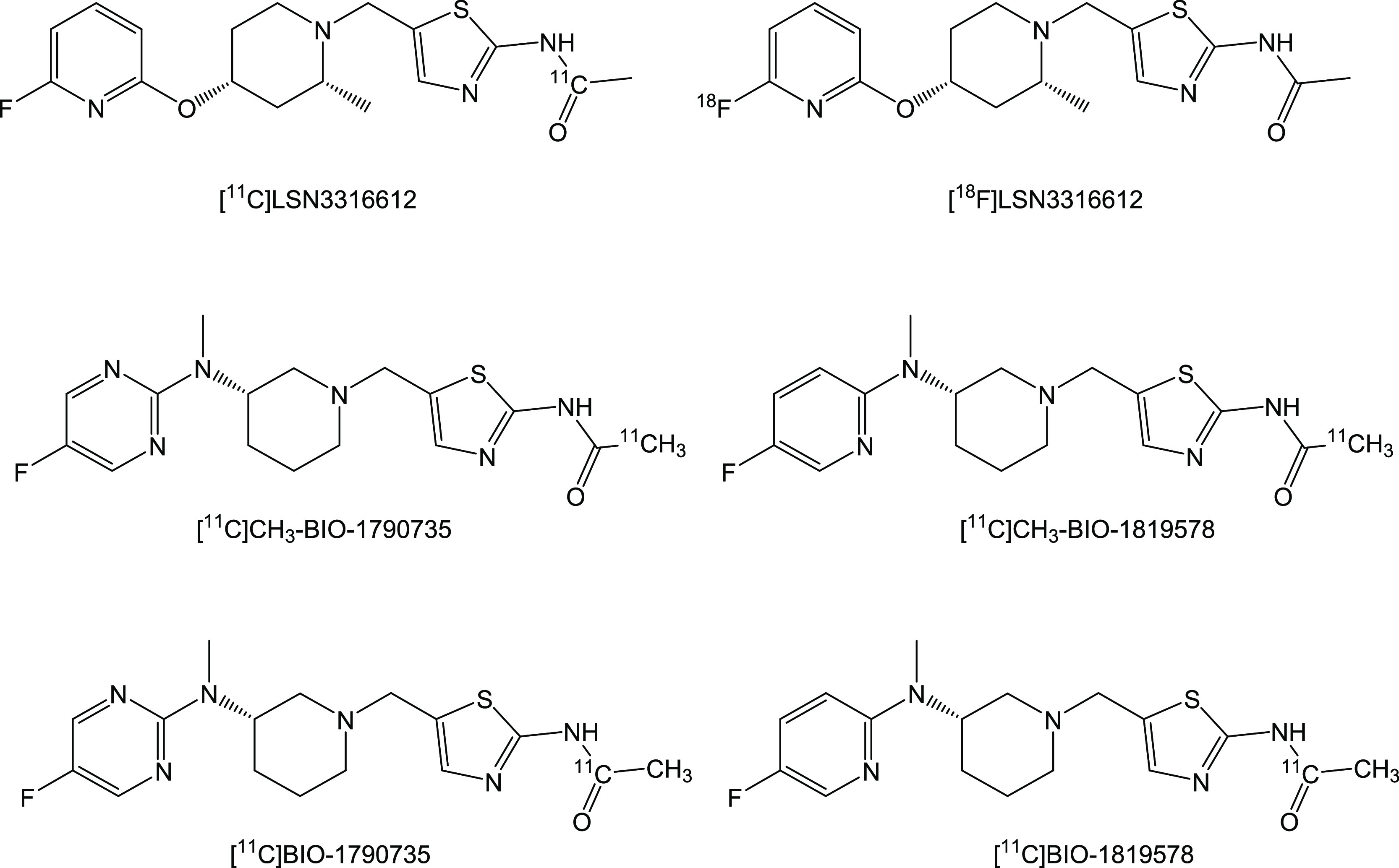
Structures of OGA radioligands.

Positron emission tomography (PET) is a sensitive and powerful molecular imaging technique for visualizing the localization of different targets in living brains.^10^ Therefore, development of a suitable PET radioligand capable of imaging OGA could provide an overview of the pathophysiological pathway of neurodegenerative diseases, provide evidence of drug-target engagement, and help in the selection of dose for therapeutic candidates. Until today, only four PET radioligands such as [^11^C]LSN3316612,^11^ [^18^F]LSN3316612,^11^ [^18^F]MK8553,^12^ and [^11^C]CH_3_-BIO-1790735^13^ have been reported to image OGA in living brains (Figure 1). [^11^C]CH_3_-BIO-1790735 is a close analogue of [^11^C]BIO-1819578, and it was labeled with C-11 using [^11^C]CH_3_I at the methyl position. The overall radiochemical yield was very low (<2%, and produced <150 MBq) to translate further to clinical PET experiments. Moreover, PET measurements with [^11^C]CH_3_-BIO-1790735 in NHP demonstrated irreversible binding kinetics. Only [^18^F]MK8553^12^ and [^18^F]LSN3316612^14^ were validated and translated further to clinical PET experiments. However, the structure of [^18^F]MK8553 has not been disclosed, and despite the initial promising characteristics of [^18^F]LSN3316612, under re-test conditions, the test–retest reliability was only modest.^14^ Moreover, the study did not measure the target occupancy (TO) of an OGA inhibitor. Based on these limitations, we sought to further develop an improved PET radioligand for imaging OGA.

**Figure 2:**
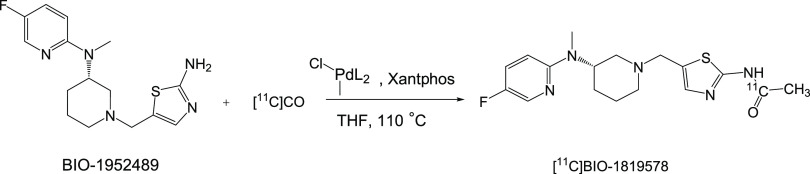
Radiosynthesis of [^11^C]BIO-1819578. Conditions: [^11^C]CO, methyl palladium(II)chloride complex, Xantphos/THF, 110 °C, 400 s.

Recently, BIOGEN developed an OGA inhibitor BIO-1819578 with a maximal inhibitory concentration (IC_50_) > 2 nM. BIO-1819578 was selected from PET tracer candidates based on a library of 366 OGA inhibitors using the IC50 for the enzyme in the nM and sub-nM range. The *K*_D_ was measured by SPR using the recombinant enzyme (6.15 nM for BIO-1819578). This ensures that the *B*_max_/*K*_D_ is greater than 10, which is an important criterion for a suitable PET radioligand development.^15^ The Ki of [^3^H]BIO-1819578 was measured using mouse brain homogenates with known OGA inhibitors such as thiamet-G and PUGNAc. The Ki values for thiamet-G and PUGNAc were identified at 0.7 and 114 nM, respectively. In this study, we focused on (i) developing an efficient synthetic method for labeling BIO-1819578 with carbon-11 using ^11^CO (Figure 2); (ii) studying the in vivo characteristics for detection of OGA enzyme in non-human primate (NHP) brain using PET; and (iii) analyzing the radiometabolism of [^11^C]BIO-1819578 in NHP blood plasma by HPLC.

## Results and Discussion

The total time for radiosynthesis including HPLC purification, SPE isolation, and formulation of [^11^C]BIO-1819578 was about 40 min. The one-step ^11^C-acylation using [^11^C]CO yielded 2569 ± 1351 MBq of the pure final product following irradiation of the cyclotron target with a beam current of 35 μA for 15–20 min. The molar activity of the produced radioligand was 14 ± 5 GBq/μmol at the time of injection to NHP. The radiochemical purity was >99% at the end of the synthesis, and the identity of the radioligand was confirmed by co-injection of the radioligand with an authentic reference standard by an HPLC equipped with both UV and radio detector. The final product [^11^C]BIO-1819578 formulated in sterile saline was found to be stable with a radiochemical purity of more than 99% for up to 60 min.

Cyclotron target-produced [^11^C]CO_2_ was utilized for the production of [^11^C]CO, which was used as the radiolabeling agent. Fully automated production of [^11^C]CO followed by trapping and incorporation of the produced [^11^C]CO into organic scaffolds was performed according to previously reported methods.^16^ Our primary focus was to synthesize [^11^C]BIO-1819578 via a ^11^C-aminocarbonylation reaction using [^11^C]CO. Initially, the synthesis was done following a published method by Pd-mediated carbonylation of methyl iodide substrate in the presence of precursor amine as the coupling partner and Xantphos, the supporting ligand for low-pressure ^11^C-carbonylation reactions.^17^ Initial experiments with methyl iodide as a substrate produced the desired product in a low radiochemical yield (RCY) of <1% and produced only 40–60 MBq of the final product, which was not enough for the in vivo evaluation of [^11^C]BIO-1819578 by using PET. Different reaction solvents such as tetrahydrofuran, DMF, DMSO, dioxane, and acetonitrile as well as different temperatures from room temperature to 120 °C were explored to optimize the reaction. No improvement of the radiochemical yield was observed.

In the next step, chloro(1,5-cyclooctadiene)methylpalladium(II) was to be explored as the preferred palladium catalyst as well as the substrate for the methyl source in this reaction.^18^ Different reaction solvents such as tetrahydrofuran, DMF, and dioxane with different amounts of precursor, reagents, and different temperatures were explored to optimize the reaction. The optimal result was achieved with CH_3_Pd(PPh_3_)_2_Cl, Xantphos, and precursor amine with appropriate amounts in THF as the solvent while heating at 110 °C for 400 s. No significant difference was observed for both THF and dioxane, which was used as a reaction solvent (Table 1). For routine production of [^11^C]BIO-1819578, THF was chosen over dioxane because of the relatively low boiling point of THF to facilitate its removal prior to injection into the HPLC for the subsequent purification step.

**Table 1:** Optimization of Radiosynthesis

precursor (mg)	alkylating agent	solvent	Temp (°C)	RCC (%)
1.0	CH_3_I	THF	RT and 100	0
2.0			60 and 120	<1
5.0			60 and 120	<1
2.0	CH_3_I	DMF	RT and 100	<1
2.0	CH_3_I	DMSO	100	0
2.0	CH_3_I	acetonitrile	RT and 80	0
1.0	CH_3_I	dioxan	120	0
2.0			120	<1
1.0	(COD)PdMeCl	THF	80	>20
			100	>25
2.0	(COD)PdMeCl	THF	110	>55
2.0	(COD)PdMeCl	THF	135	>50
5.0	(COD)PdMeCl	THF	110	>55
2.0	(COD)PdMeCl	dioxan	110	>55
5.0	(COD)PdMeCl	dioxan	110	>50

**Figure 3:**
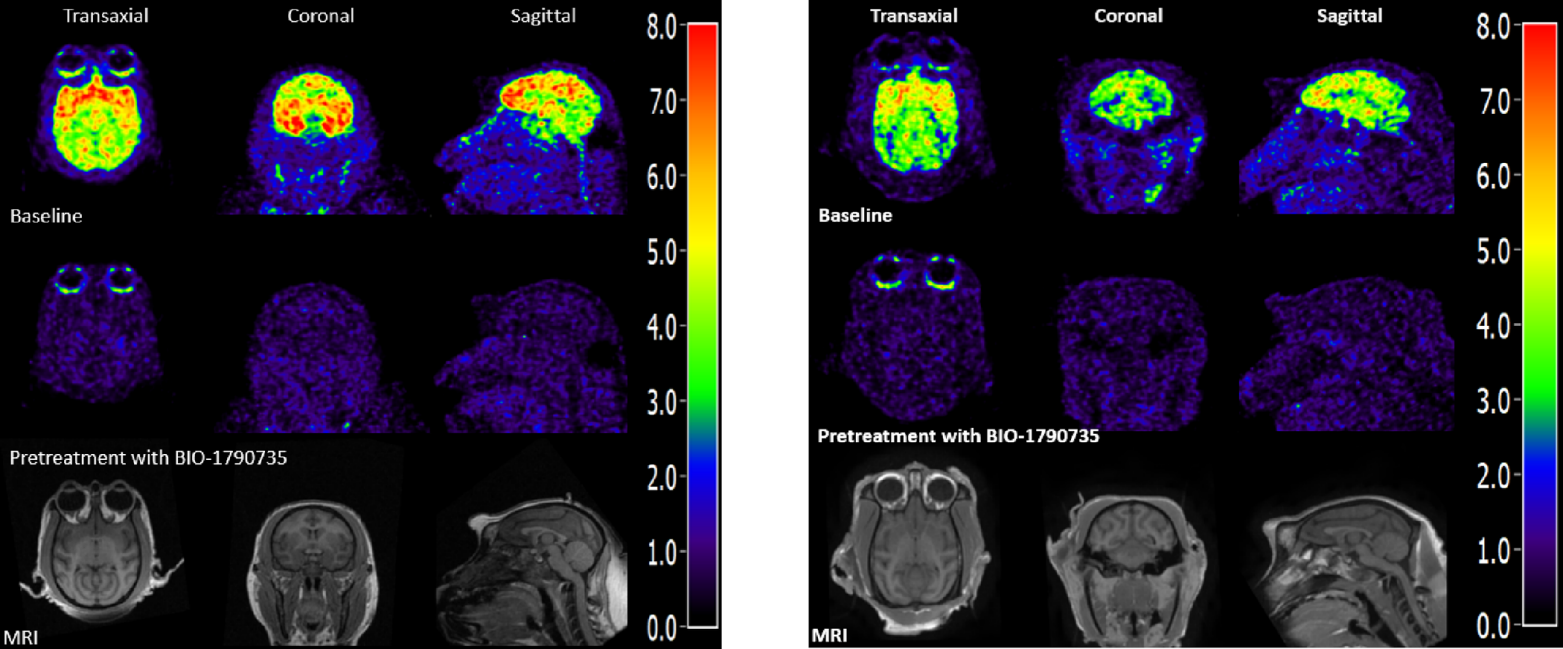
(A) PET images of [^11^C]BIO-1819578 co-registered with MRI and averaged between 60 and 90 min in the transaxial (left), coronal (middle) and sagittal (right) projections at baseline (upper) and blocking with BIO-1790735 (middle). Anatomical reference T1w MRI (bottom). (B) PET images of [^11^C]BIO-1819578 co-registered with MRI and averaged between 60 and 90 min in the transaxial (left), coronal (middle) and sagittal (right) projections at baseline (upper) and blocking with thiamet-G (middle). Anatomical reference T1w MRI (bottom).

**Figure 4:**
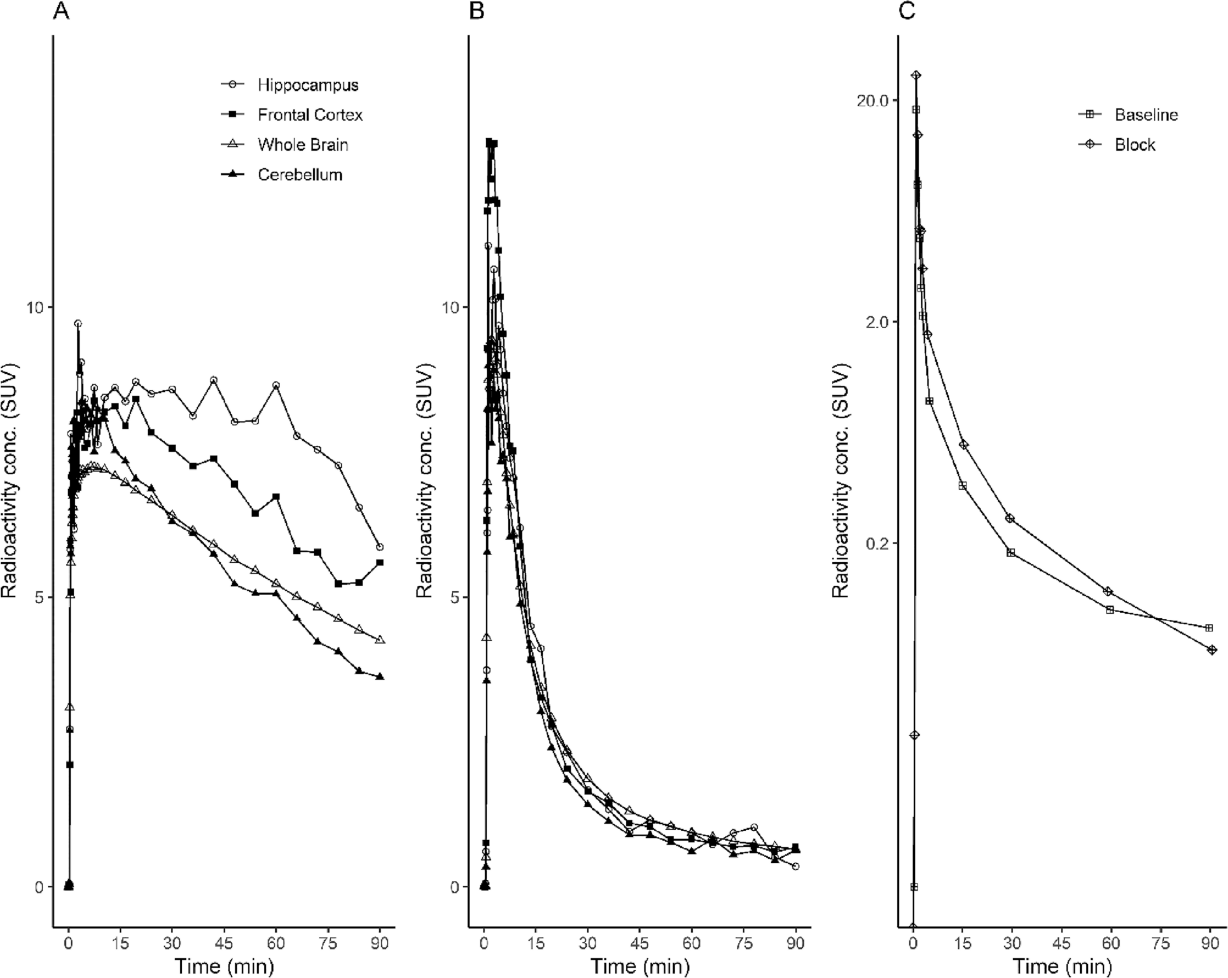
Time–activity curves represent the concentration of radioactivity in the NHP brain (A) at baseline and (B) after pretreatment with BIO-1790735 and unchanged [^11^C]BIO-1819578 in plasma (C) after intravenous injection of [^11^C]BIO-1819578 into a non-human primate. The plasma curve represents the radiometabolite-corrected arterial input function.

**Figure 5:**
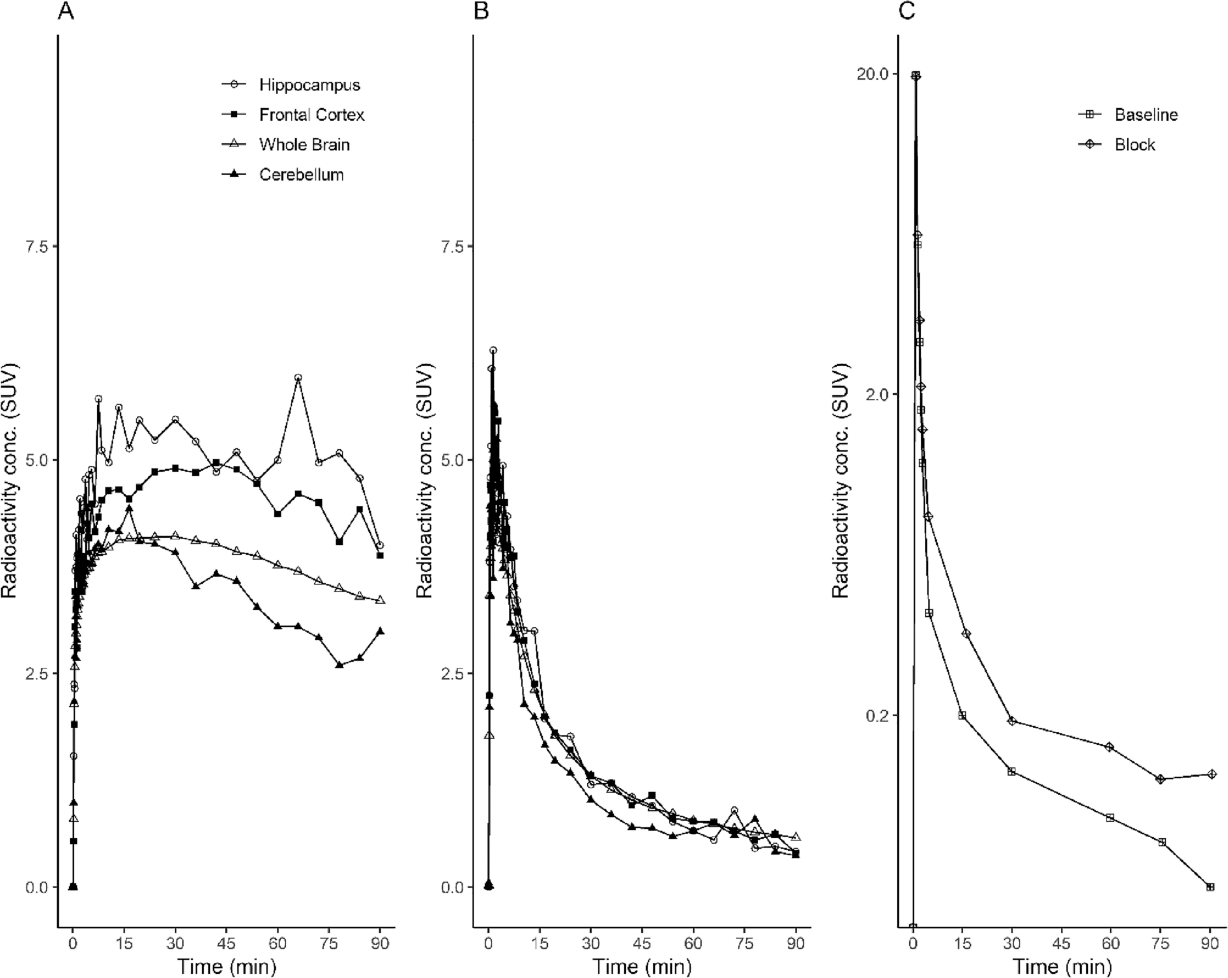
Time–activity curves representing the concentration of radioactivity in the NHP brain (A) at baseline and (B) after pretreatment with thiamet-G and unchanged [^11^C]BIO-1819578 in plasma (C) after intravenous injection of [^11^C]BIO-1819578 into a non-human primate. The plasma curve represents the radiometabolite-corrected arterial input function.

At the time of injection, the injected radioactivity of [^11^C]BIO-1819578 was 86 ± 9 MBq and the injected mass was 2.5 ± 1.1 μg. The summated PET images for both baseline and the two blocking studies as well as T1w MRI for anatomical reference are shown in Figure 3A,B. The whole brain uptake of [^11^C]BIO-1819578 was over 7 SUV at peak for the baseline condition. Initially, a rapid increase in radioligand uptake was observed across the brain, with varying SUV depending on different brain regions. The cerebellum showed the lowest peak SUV with values ranging from 4 to 6 g/mL, while the hippocampal peak uptake was higher with a range of 5–9 g/mL. The tracer showed washout in all brain regions, demonstrating reversible kinetics for the tracer (Figures 4A and 5A). After pretreatment with either BIO-1790735 (1 mg/kg) or the well-known brain barrier-penetrating OGA inhibitor thiamet-G (10 mg/kg), the tracer uptake was substantially decreased, as indicated in Figures 4B and 5B, respectively. Figures 4C and 5C show that the plasma parent tracer concentration remains similar for both baseline and blocking scans. The reduction values of late whole brain SUV were 82 and 83% for blocking with BIO-735 and thiamet-G, respectively, demonstrating target engagement for [^11^C]BIO-1819578 in the NHP brain.

**Figure 6:**
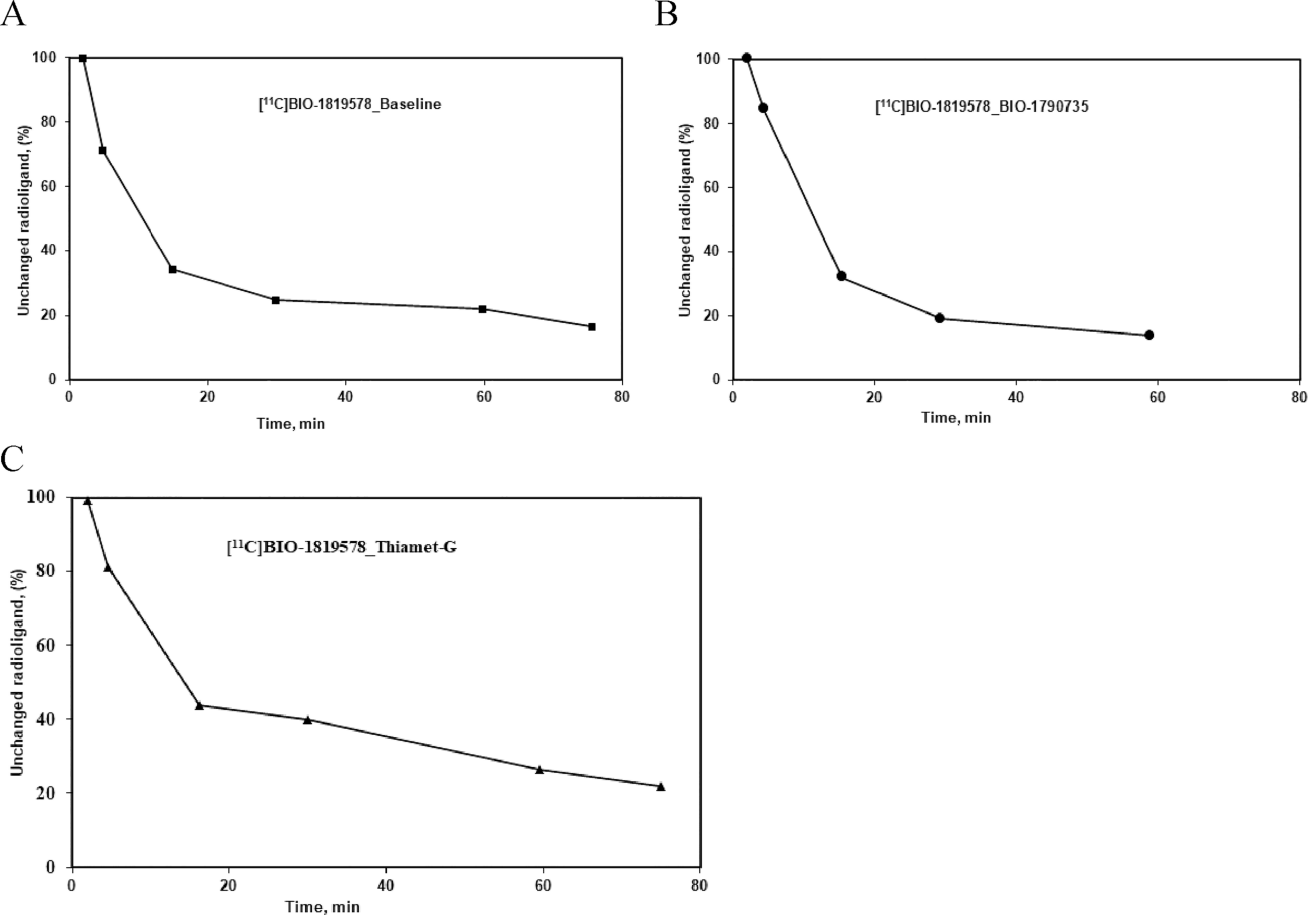
Radiometabolite analysis during the course of the PET measurements. The in vivo metabolism of [^11^C]BIO-1819578 is shown as the relative plasma composition at baseline condition (left), after pretreatment with BIO-1790735 (middle), and after pretreatment with thiamet-G (right).

More than 95% of the radioactivity was recovered from plasma into acetonitrile after deproteinization. In the HPLC analysis of plasma following injection, [^11^C]BIO-1819578 was eluted from the HPLC column at 5.4 min retention time. The parent compound was more abundant at 5 min, representing approximately 90%, and it decreased to about 15% at 60 min for PET at the baseline condition (Figure 6 left) as well as after pretreatment with BIO-1790735 (Figure 6 middle). Meanwhile, abundance of the parent compound decreased to about 30% at 60 min for PET after pretreatment with thiamet-G (Figure 6 right). A few more polar radiometabolite peaks were observed, which were eluted from the HPLC column before the parent peak (Figure 6A). The identity of [^11^C]BIO-1819578 was confirmed by co-injection with the non-radioactive BIO-1819578.

## Materials and Methods

### General

Both the precursor ((*S*)-5-((3-((5-fluoropyridin-2-yl)(methyl)amino)piperidin-1-yl)methyl)thiazol-2-amine) and the non-radioactive reference standard ((*S*)-*N*-(5-((3-((5-fluoropyridin-2-yl)(methyl)amino)piperidin-1-yl)methyl)thiazol-2-yl)acetamide (BIO-1819578)) were synthesized by BIOGEN MA Inc., USA. All other chemicals and reagents were bought from commercial sources. Solid-phase extraction (SPE) cartridges SepPak C18 Plus were purchased from Waters (Milford, MA, USA). The C-18 Plus cartridge was activated using EtOH (10 mL) followed by sterile water (10 mL). Liquid chromatography analysis (LC) was performed with a Merck-Hitachi gradient pump and a Merck-Hitachi, L-4000 variable wavelength UV-detector. Radiosynthesis and purification of [^11^C]BIO-1819578 were performed using a fully automated synthesis module The TracerMaker (Scansys Laboratorieteknik, Denmark).

### Synthesis of [^11^C]Carbon Monoxide ([^11^C]CO)

Synthesis of [^11^C]carbon monoxide ([^11^C]CO) was performed following a previously published method with modification.^19^ No carrier-added [^11^C]CO_2_ was produced via a ^14^N(p,α)^11^C nuclear reaction on a mixture of nitrogen and oxygen gas (0.5% oxygen) and 16.5 MeV protons produced by GEMS PET trace cyclotron (GE, Uppsala, Sweden). At the end of bombardment (EOB), the target content was delivered to the [^11^C]CO synthesizer prototype, where [^11^C]CO_2_ was trapped on a silica gel (10 mg, 60 Å, 60–100 mesh) trap immersed in liquid nitrogen. Concentrated [^11^C]CO_2_ was released from the trap by thermal heating followed by the reduction online to [^11^C]CO using a pre-heated (Carbolite oven, 850 °C) quartz glass column (6 × 4 × 180 mm: o.d. × i.d. × length) filled with molybdenum powder (1.5 g, <150 μm, 99.99% trace metal basis, Sigma Aldrich). Produced [^11^C]CO was trapped and concentrated on a silica gel (10 mg, 60 Å, 60–100 mesh) trap immersed in liquid nitrogen. Unreacted [^11^C]CO_2_ was subsequently removed by a sodium hydroxide-coated silica (0.2 g, Ascarite II, 20–30 mesh) trap (30 mm 1/8” SS tube). After completing the entrapment, the trap was heated by thermal heating to release the [^11^C]CO for further use.

### Synthesis of [^11^C]BIO-1819578

**Figure 7:**
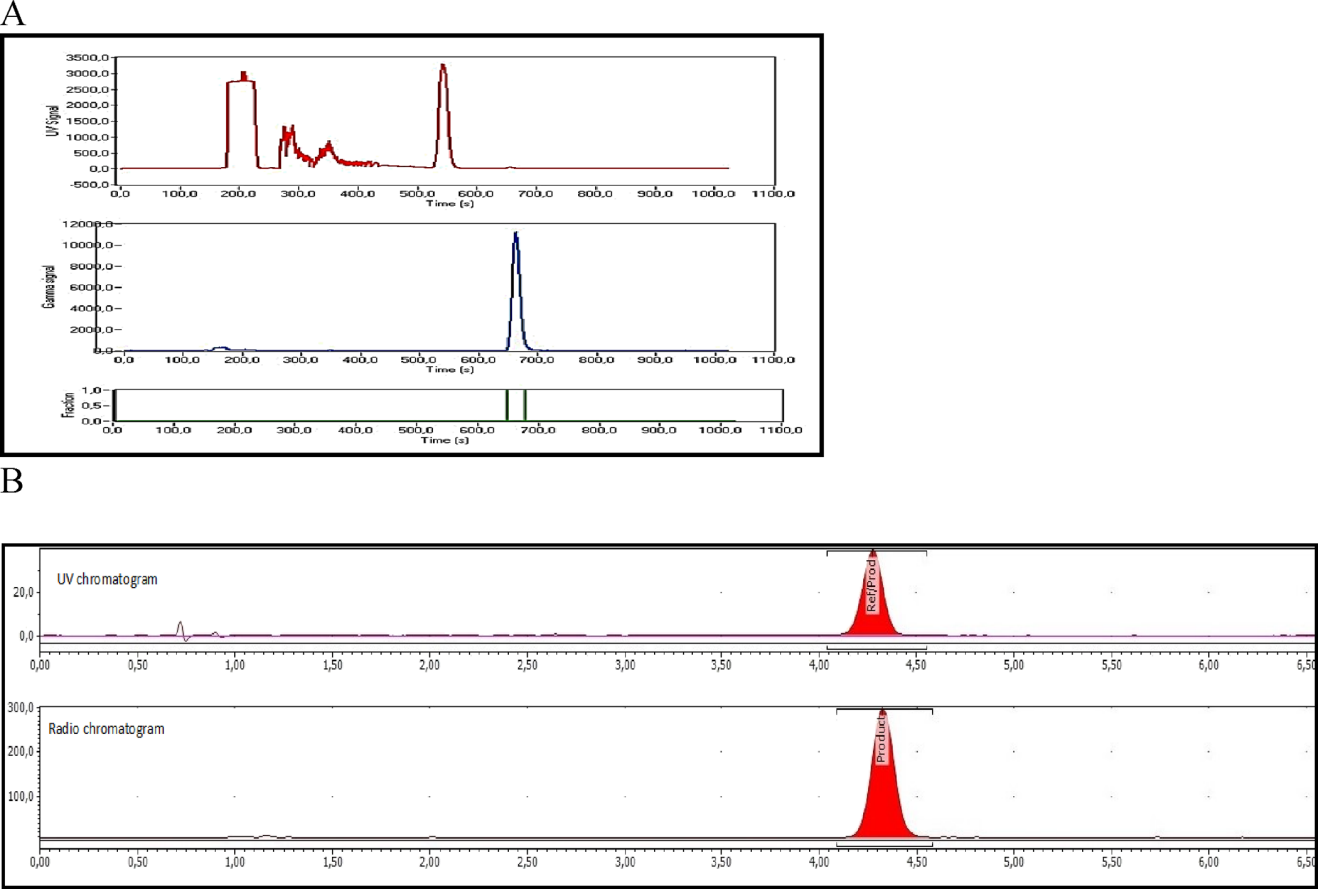
(A) HPLC chromatogram (upper: UV and bottom: radio chromatogram) of the semi-preparative purification of [^11^C]BIO-1819578. (B) HPLC chromatogram of the analysis of [^11^C]BIO-1819578 co-injected with the cold reference standard PF-06885190. The upper represents the radio-chromatogram of [^11^C]BIO-1819578, and the bottom represents the UV chromatogram of BIO-1790735.

[^11^C]BIO-1819578 was obtained by trapping [^11^C]CO at room temperature in a reaction vessel containing a mixture of the amine precursor (BIO-1952489, 2 mg, 0.006 mmol), methyl palladium(II)chloride complex (8,0 mg, 0.03 mmol), and Xantphos (12 mg, 0.022 mmol) in THF (400 μL) followed by heating at 110 °C for 400 s. After the synthesis, DMSO (500 μL) was added to the crude reaction mixture, and THF was evaporated off with a flow of helium. The residue was diluted with sterile water (2 mL) and was injected into the HPLC injection loop for purification. The loop was connected to the built-in high-performance liquid chromatography (HPLC) system equipped with a semi-preparative reverse phase (RP) ACE column (C18, 10 × 250 mm, 5 μm particle size). The column outlet was connected with a Merck Hitachi UV detector (λ = 254 nm) (VWR, International, Stockholm, Sweden) in series with a GM-tube (Carroll-Ramsey, Berkley, CA, USA) used for radioactivity detection. A mixture of acetonitrile (15%) and H_3_PO_4_ (0,01 M) (85%) with a flow rate of 4 mL/min was used as the HPLC isocratic mobile phase. The radioactive fraction corresponding to the desired product [^11^C]BIO-1819578 was eluted with a retention time (*t*_R_) 10–11 min (Figure 7A) and was diluted with sterile water (50 mL). The resulting mixture was passed through a preconditioned SepPak tC18 plus cartridge. The cartridge was washed with sterile water (10 mL), and the corresponding isolated [^11^C]BIO-1819578 was eluted with 1 mL of ethanol into a sterile vial containing sterile saline (9 mL) and sodium ascorbate (5 mg). The formulated product was then sterile-filtered through a Millipore Millex GV filter unit (0.22 μm) for further use.

### Quality Control and Molar Activity (MA) Determination

The radiochemical purity and stability of [^11^C]BIO-1819578 were determined using an analytical HPLC coupled with an analytical XBridge column (C18 5 μm, 4.6 × 150 mm particle size), Merck-Hitatchi L-7100 Pump, L-7400 UV detector, and GM-tube for radioactivity detection (VWR International). A mixture of acetonitrile (30%) and HCO_2_NH_4_ (0.1 M aq. solution) (70%) with a flow rate of 2 mL/min was used as an HPLC isocratic mobile phase. The HPLC liquid flow was monitored with a UV absorbance detector (ƛ = 254 nm) coupled to a radioactive detector (BETA-flow, Beckman, Fullerton, CA). [^11^C]BIO-1819578 was eluted with a retention time (*t*_R_) 3.5–4.5 min, and the run time of the HPLC program was 7 min (Figure 7B). The identity of the radiolabeled compounds was confirmed by HPLC with the co-injection of the corresponding authentic reference standard.

The MA was determined by analytical HPLC following the method described elsewhere.^20^

### Study Design in Non-human Primates, PET Experimental Procedure, and Quantification

Two cynomolgus monkeys (*Macaca fascicularis*, NHP), one female (7.3 kg) and one male (6.8 kg), were studied in two different experimental days for a total of four PET measurements. Both NHPs underwent two PET measurements on the same day. The first PET measurement was performed with [^11^C]BIO-1819578 followed by the second PET measurement after pretreatment with either BIO-1790735 (1 mg/kg) or thiamet-G (10 mg/kg), 3 h after the first one. Both NHPs were supplied by the Astrid Fagraeus Laboratory of the Swedish Institute for Infectious Disease Control (SMI), Solna, Sweden. The study was approved by the Animal Ethics Committee of the Swedish Animal Welfare Agency and was performed according to “Guidelines for Planning, Conducting and Documenting Experimental Research” (Dnr 10367-2019) of the KI as well as the “Guide for the Care and Use of Laboratory Animals”.^21^ Anesthesia was induced by repeated intramuscular injections of a mixture of ketamine hydrochloride (3.75 mg/kg/h Ketalar Pfizer) and xylazine hydrochloride (1.5 mg/kg/h Rompun Vet., Bayer). To fix the position of the head of the NHP during the course of whole PET measurement, a prototype device was used.^22^ Body temperature was maintained using a Bair Hugger-Model 505 (Arizant Health Care Inc., MN, USA) and monitored using an oral thermometer. ECG, heart rate, respiratory rate, and oxygen saturation were continuously monitored throughout the experiments, and blood pressure was monitored every 15 min.

A high-resolution research tomograph (HRRT) (Siemens Molecular Imaging) was used to perform all the PET measurements of this study. The corresponding in-plane resolution with OP-3D-OSEM PSF was 1.5 mm full width at half-maximum (FWHM) in the center of the field of view (FOV) and 2.4 mm at 10 cm off-center directions.^23^ List-mode data were acquired continuously for 93 min immediately after intravenous injection of radioligands. Images were reconstructed by the ordinary Poisson-3D-ordered subset expectation maximization (OP-3D-OSEM) algorithm with 10 iterations and 16 subsets including modeling of the point spread function (PSF). A 6 min transmission scan was performed using a single ^137^Cs source, prior each PET acquisition for attenuation and scatter correction. Brain magnetic resonance imaging was performed on a 1.5-T GE Signa system (General Electric, Milwaukee, WI, USA). A T1-weighted image was obtained for co-registration with PET and delineation of anatomic brain regions. The T1 sequence was a 3D spoiled gradient recalled (SPGR) protocol with the following settings: repetition time (TR) 21 ms, flip angle 35°; FOV 12.8; matrix 256 × 256 × 128; 128 × 1.0 mm slices; 2 NEX. The sequence was optimized for trade-off between a minimum of scanning time and a maximum of spatial resolution and contrast between gray and white matter.

Before delineation of regions of interest (ROIs), the orientation of the brain was spatially normalized by having the high-resolution T1-weighted magnetic resonance images reoriented according to the line defined by the anterior and posterior commissures being parallel to the horizontal plane and the interhemispheric plane being parallel to the sagittal plane. The T1-weighted MR images were then resliced to the resolution of the HRRT PET system, 1.219 × 1.219 × 1.219 mm. The standardized T1-weighted MR images were used as an individual anatomical template for the monkey.

Both NHPs underwent with an i.v. injection of the corresponding radioligand [^11^C]BIO-1819578. The regions of interest (ROIs) were delineated manually on MRI images of each NHP for the whole brain, putamen, caudate, frontal cortex, occipital cortex, hippocampus, cerebellum, and thalamus. The summed PET images of the whole duration were co-registered to the MRI image of the individual NHP. After applying the co-registration parameters to the dynamic PET data, the time–activity curves of brain regions were generated for each PET measurement. The average standardized uptake value (SUV) was calculated for each brain region using the following equation:
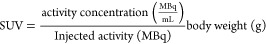


### Radiometabolite Analysis

Analysis of radiometabolites in NHP blood plasma was performed according to a previously published method.^24^ A reverse-phase HPLC with a UV absorbance detector (ƛ = 254 nm) coupled to a radioactive detector was used to determine the percentages of radioactivity corresponding to [^11^C]BIO-1819578 and its radioactive metabolites during the course of whole PET measurement. Arterial blood samples (0.7–1.5 mL) were obtained from the monkey at different time points such as 2, 5, 15, 30, 60, and 75 min after injection of [^11^C]BIO-1819578. Plasma was separated from the collected blood by centrifuging at 4000 rpm for 2 min. The obtained plasma was diluted with 1.4 times volume of acetonitrile followed by centrifugation at 6000 rpm for 4 min. The extract was separated from the pellet and was diluted with water (3 mL) before injecting into the HPLC. The HPLC system was coupled to an Agilent binary pump (Agilent 1200 series), which was connected to a manual injection valve (7725i, Rheodyne), 5.0 mL loop, and a radiation detector (Oyokoken, S-2493Z) housed in a shield of 50 mm-thick lead. A semipreparative reverse phase ACE 5 μm C18 HL column (250 × 10 mm) was used to achieve the chromatographic separation of the radiometabolites from the unchanged [^11^C]BIO-1819578 by gradient elution. Acetonitrile (A) and 0.1 M ammonium formate (B) were used as the mobile phase at 5.0 mL/min, according to the following program: 0–4.0 min, (A/B) 40:60 → 90:10 v/v; 4.0–6.0 min, (A/B) 90:10 v/v. The radioactive peak corresponding to the [^11^C]BIO-1819578 eluted from the HPLC column was integrated, and the area was expressed as a percentage of the sum of the areas of all detected radioactive compounds. To calculate the recovery of radioactivity from the system, an aliquot (2 mL) of the eluate from the HPLC column was measured and divided with the amount of total injected radioactivity.

## Conclusions

The present study demonstrated that the radioligand [^11^C]BIO-1819578 was efficiently labeled with carbon-11 at the carbonyl position using [^11^C]CO. PET measurements in two cynomolgus monkeys showed high brain uptake, which was significantly decreased after pretreatment with OGA inhibitor BIO-1790735 or thiamet-G, indicating specific binding. On the basis of these data, [^11^C]BIO-1819578 merits further evaluation with full quantification. The results demonstrate that [^11^C]BIO-1819578 is a potential PET radioligand for imaging OGA in human brain.
